# Mutations in *Mycobacterium tuberculosis* Isolates with Discordant Results for Drug-Susceptibility Testing in Peru

**DOI:** 10.1155/2020/8253546

**Published:** 2020-04-06

**Authors:** L. Solari, D. Santos-Lazaro, Z. M. Puyen

**Affiliations:** ^1^Instituto Nacional de Salud, Lima, Peru; ^2^Escuela de Medicina, Universidad Peruana de Ciencias Aplicadas, Lima, Peru

## Abstract

Evaluation of resistance to antituberculosis drugs is routinely performed with genotypic or phenotypic methods; however, discordance can be seen between these different methodologies. Our objective was to identify mutations that could explain discordant results in the evaluation of susceptibility to rifampicin and isoniazid between molecular and phenotypic methods, using whole genome sequencing (WGS). Peruvian strains showing sensitive results in the GenoType MTBDR*plus* v2.0 test and resistant results in the proportions in the agar-plaque test for isoniazid or rifampin were selected. Discordance was confirmed by repeating both tests, and WGS was performed, using the Next Generation Sequencing methodology. Obtained sequences were aligned “through reference” (genomic mapping) using the program BWA with the algorithm “mem”, using as a reference the genome of the *M. tuberculosis* H37Rv strain. Discordance was confirmed in 14 strains for rifampicin and 21 for isoniazid, with 1 strain in common for both antibiotics, for a total of 34 unique strains. The most frequent mutation in the rpoB gene in the discordant strains for rifampicin was V170F. The most frequent mutations in the discordant strains for isoniazid were katG R463L, kasA G269S, and Rv1592c I322V. Several other mutations are reported. This is the first study in Latin America addressing mutations present in strains with discordant results between genotypic and phenotypic methods to rifampicin and isoniazid. These mutations could be considered as future potential targets for genotypic tests for evaluation of susceptibility to these drugs.

## 1. Introduction

Peru is one of the 30 countries in the world with the highest burden of multidrug-resistant tuberculosis (MDR-TB). The public health system provides free diagnosis and drug-susceptibility testing with phenotypic (proportions in 7H10)[1] and molecular methods (Genotype, Hain Lifesciences (Genotype™)) [2] for first- and second-line drugs. Molecular diagnosis of drug resistance has been shown to significantly reduce the time to diagnosis, most of all to isoniazid and rifampicin, the pillars of antituberculosis regimens, and thus contribute to improve the clinical outcomes [3] However, the reference standard continue to be phenotypic methods, such as MGIT 960 and proportions in agar plaque, both currently used at the National Reference Laboratory of Mycobacteria of Peru.

The Genotype method is based on the identification of mutations of the mutations of the rpoB gene in the Rifampicin-resistance determining region (RRDR), an 81 base-pair segment of the gene. Mutations in the katG and inhA genes are identified to diagnose resistance to isoniazid. However, this assay identifies the most frequent mutations but not every possible mutation conferring resistance to these drugs. There could be mutations unidentified by these tests (“noncanonical” mutations), and so the test would be reported as susceptible [4]. Noteworthy, if phenotypic methods were used, a resistant pattern would be detected. There are reports postulating novel mutations to explain discrepancies between molecular and phenotypic methods [5, 6], as these could explain the discordant results. Whole Genome Sequencing (WGS) is a technology that can identify new sequences that could explain certain traits related to resistance, and could currently be used to investigate the genome of *Mycobacterium tuberculosis* strains showing this kind of discordant results [7].

However, not all existing mutations determine resistance, so it is important to consider existing evidence about their association with drug resistance. The World Health Organization has issued recommendations on their interpretation [8]. Knowledge on this topic continues to evolve, and it is very important to generate additional information about the presence of these novel mutations. Our aim was to identify mutations that could explain discordant results in the evaluation of susceptibility to rifampicin and isoniazid between molecular and phenotypic methods, using whole genome sequencing. This information could contribute in the future to develop more comprehensive diagnostic devices for evaluation of drug resistance in our country.

## 2. Materials and Methods

### 2.1. Strains

The National Reference Laboratory for Mycobacteria of the National Institute of Health of Peru performs evaluation of first-line drug-resistance with Genotype™ since 2011. Only samples showing any kind of resistance undergo phenotypic drug resistance evaluation with the proportions method in Middlebrook 7H10 Agar for 11 drugs, including isoniazid and rifampicin. However, between 2013 and 2015, a national survey was performed, and samples were analysed with both methods in parallel. We selected strains collected in that period showing a discordant result between both methods (sensitive in the GenoType MTBDR*plus* v2.0 test and resistant in the proportions in agar-plaque test) for isoniazid or rifampicin.

### 2.2. Laboratory Procedures

*Mycobacterium tuberculosis* strains kept at −80°C available at the biobank of this laboratory were reactivated in Middelbrook 7H9 broth for 7 days. An aliquot of 0.5 mL was then transferred to Löwenstein–Jensen media for further growth.

According to Hofmann-Thiel et al.'s recommendations [9], we repeated the Genotype® MTBDR*plus*, Hain LifeScience, Nehren, Germany v2.0 test and additionally the proportions in the agar-plaque test to confirm the results. GenoType MTBDR*plus* v2.0 (http://www.hain-lifescience.com) was performed following the manufacturer´s indications. The GenoLyse v1.0 kit was used for DNA extraction, and the DNA amplification and hybridization was performed using the Genotype MTBDR*plus* v2.0 probes for identification of mutations in the *rpoB*, *katG*, and *inhA* genes.

For the proportions in the agar-plaque test, the colonies were transferred from the Löwenstein–Jensen media to Middlebrook 7H9 for 7 days and subsequently to Middlebrook 7H10 for 21 days. The plaques contained rifampicin at a minimum inhibitory concentration of 1.0 *μ*g/mL and isoniazid at 2 minimum inhibitory concentrations: 0.2 *μ*g/mL and 1.0 *μ*g/mL. For the interpretation, if the critical proportion was higher than 1%, the strain was considered resistant, and if it was less than 1%, it was considered sensible [10, 11]. Only strains showing persisting discordance after repeating the tests were included for analysis of WGS. Genomic DNA was extracted from these strains using the “GenJet Genomic DNA purification” (http://www.thermofisher.com) kit, according to the manufacturer's recommendations. Then, the double-stranded DNA concentration was quantified, by fluorescence, using the Qubit 2.0 fluorometer kit (Invitrogen, Carlsbad, CA, USA). Whole Genome Sequencing was performed using paired-end of 2 × 250 and 2 × 300 pb of the Nextera XT kit with the system NGS illumina MiSeq (Illumina Inc., San Diego, CA, USA). The MiSeq Reagent Kit v3 (600 Cycles) was used as recommended (https://www.illumina.com).

### 2.3. Bioinformatic Analysis

Quality control was performed to the obtained reads using the FastQC program v0.11.8 [12]. The estimation of the purity of the reads and of the purity of the sequencing was determined with the programs Kraken2 v2.0.7 [13] and Bracken v2.2 [14]. The reads were then cleaned using the program Trimmomatic v0.38 [15].

The sequences obtained were aligned “through reference” (genomic mapping) using the program BWA [16], with the algorithm “mem”, using as a reference the genome of the strain *M. tuberculosis* H37Rv (GenBank, Access number: NC_000962.3). Identification of duplicates and the final ordering was performed with the tool Picard v2.18.25 (http://broadinstitute.github.io/picard).

Finally, we report the sequences of the *rpoA, rpoB*, *and rpoC* genes for isolates with discordant results for rifampicin and the sequences of the *katG* and i*nhA* genes for isolates with discordant results for isoniazid. In order to classify the mutations as having low, median, or high probability of conferring drug resistance (as not all mutations are necessarily associated with resistance), we followed the recommendations of the World Health Organization [9] and complemented with information found in other publications. For rifampicin resistance, we report mutations both according to the consensus proposed by Telenti et al. [17], as well as to Andre et al.'s notation [18]. For the descriptive statistical analysis, Stata ver. 15 was used.

## 3. Results

### 3.1. Strains

From 2013 to 2015, we were able to confirm discordant results (reported as sensitive for rifampicin or isoniazid with the GenoType MTBDR*plus* v2.0 and resistant to the same drug with the proportions method in Middlebrook 710 Agar) in 36 strains, 16 for rifampicin and 21 for isoniazid (1 sample was discordant for both drugs), which were processed for whole genome sequencing.  Table 1 shows the main characteristics of the corresponding patients. Noteworthy, this accounts for approximately 0.5% of the 7194 valid results for this period.

### 3.2. Rifampicin

**Figure 1:**
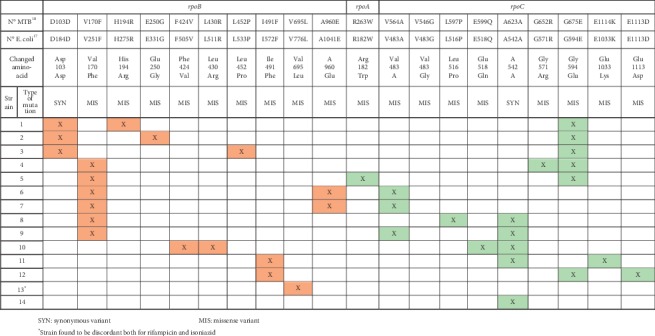
Mutations present in genes *rpoB*, *rpoA*, and *rpoC* in strains with discordant results between genotypic and phenotypic methods for rifampicin. SYN: synonymous variant. MIS: missense variant. ^*∗*^Strain found to be discordant both for rifampicin and isoniazid.

Sixteen strains were found to have discordant results for rifampicin in the phenotypic and genotypic methods. Of these, we were able to recover and perform WGS in 14. All strains had missense mutations in *rpoB*. The most frequent mutation was V170F, found in 6 samples.  Figure 1 shows all mutations present in the genes *rpoB* (probably the ones determining resistance), *rpoA*, and *rpoC* (probably compensatory mutations) in these 14 strains.

### 3.3. Isoniazid

**Figure 2:**
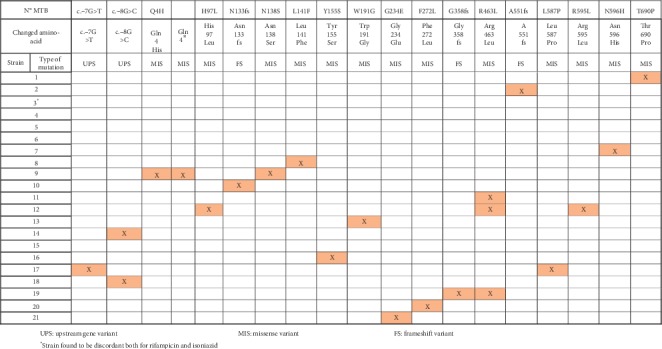
Mutations present in the katG gene in strains with discordant results between genotypic and phenotypic methods for isoniazid. UPS: upstream gene variant. MIS: missense variant. FS: frameshift variant. ^*∗*^Strain found to be discordant both for rifampicin and isoniazid.

**Figure 3:**
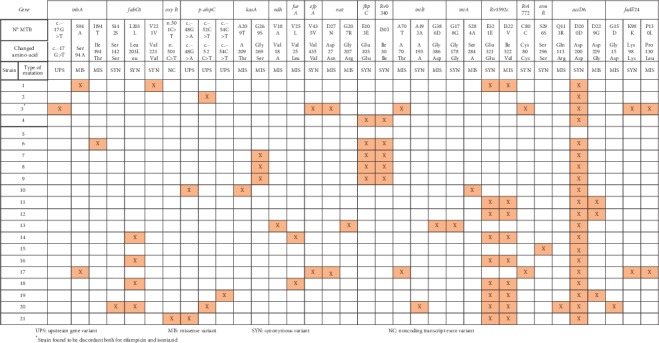
Mutations present in diverse genes in strains with discordant results between genotypic and phenotypic methods for isoniazid. UPS: upstream gene variant. MIS: missense variant. SYN: synonymous variant. NC: noncoding transcript exon variant. ^*∗*^Strain found to be discordant both for rifampicin and isoniazid.

Twenty-one strains were found to have discordant results for isoniazid between the phenotypic and genotypic methods. We were able to recover and perform WGS in all of them. The mutations found were very diverse in gene *katG,* (Figure 2) being the most frequent missense mutation R463L, present in 3 samples. We did not find the following mutations in the *katG* gene: c.−10A > C, P92S, A109T, K154T, P235S, T271A, G307V, S315T, S315N, P365P, T380I, R396L, D419, P432L, A478A, A551A, N660T, N660D, E709G, and G177S.  Figure 3 shows all mutations present in the other genes (*inhA, fabG1, oxyR, p-ahpC, kasA, ndh, furA, efpA, nat, fbpC, Rv0340, iniB, iniA, Rv1592, Rv1772, srmR, accD6,* and *fadE24*) that could be related to isoniazid resistance, the most frequent mutations being missense: *kasA* G269S in 3 strains and *Rv1592c* I322V in 9 samples. Interestingly, we were not able to find mutations other than synonymous in strains 4 and 15, and in strain 5, we were not able to find any mutation.

## 4. Discussion

This is the first study in Latin America addressing mutations present in a considerable group of strains of *Mycobacterium tuberculosis* that show sensitivity to the most important first-line antituberculosis drugs (rifampicin and isoniazid) in genotypic tests and resistance to the same drugs (discordance) in phenotypic tests, using Whole Genome Sequencing to find “noncanonical” mutations. Indeed, we were able to find missense mutations present in these discordant strains that could explain the resistance to rifampicin (*rpoB* V170F, I491F and others) and to isoniazid (*katG* R463L, *kasA G269S, Rv1592c I322V*, and others).

This is a relevant finding, because these could be considered potential targets for the development of diagnostic devices for drug resistance in the future [19]. Noteworthy, some of the mutations, such as R463L in *katG*, have also been signaled by other groups, highlighting their potential relevance to confer resistance [20]. Others are reported for the first time in this publication, some of which could be incidental findings, not necessarily conferring resistance to antituberculosis drugs, and others could be confirmed through future research. Although we have focused on missense and frame shifting mutations, we have reported upstream and synonymous mutations as well, to provide a complete oversight of the detected changes. All this will contribute to the available information about the genomic variants of the *Mycobacterium tuberculosis* and could have other potential applications, such as in the study of epidemiological dynamics and spreading of drug-resistant strains.

We have to acknowledge some issues that could limit the external validity of our results. In the first place, the samples were taken in the years 2013–2015, so we would have to be cautious before considering these strains are representative of the strains currently circulating in our territory. In the second place, we have to consider that, although there are some initiatives for approaching consensus in the mutation conferring resistance, this is a new field and the information is still preliminary. Hence, we cannot ensure that the mutations we have reported are really associated with resistance. The ideal approach, though resource-consuming, would be to produce these mutations in sensitive organisms to evaluate if they actually become resistant [21].

## 5. Conclusions

In *Mycobacterium tuberculosis* strains showing discordant results between phenotypic and genotypic diagnostic tests for evaluation of drug resistance to rifampicin and isoniazid, we were able to report several mutations identified through WGS. Some of these, in particular *rpoB* V170F and I491F for rifampicin and *katG* R463L, *kasA* G269S, and Rv1592c I322V for isoniazid, could be considered in the future for development of local genotypic diagnostic tests for diagnosis of drug resistance.

## Figures and Tables

**Table 1:** Characteristics of included patients.

Characteristic	Number (percentage) (*n* = 36)
Sex, male	29 (80.6%)

Age (median, interquartile range)	28.9 (24–38)

Treatment history	
No previous treatment	17 (47.2%)
Previously treated	8 (22.2%)
No information	11 (30.5%)

Area of residence	
Lima (capital)	30 (83.3%)
Province	6 (16.7%)

## Data Availability

The data on Whole Genome Sequencing used to support the findings of this study are available from the corresponding author upon request.
